# Natural history of limb girdle muscular dystrophy R9 over 6 years: searching for trial endpoints

**DOI:** 10.1002/acn3.774

**Published:** 2019-05-16

**Authors:** Alexander P. Murphy, Jasper Morrow, Julia R. Dahlqvist, Tanya Stojkovic, Tracey A. Willis, Christopher D. J. Sinclair, Stephen Wastling, Tarek Yousry, Michael S. Hanna, Meredith K. James, Anna Mayhew, Michelle Eagle, Laurence E. Lee, Jean‐Yves Hogrel, Pierre G. Carlier, John S. Thornton, John Vissing, Kieren G. Hollingsworth, Volker Straub

**Affiliations:** ^1^ The John Walton Muscular Dystrophy Research Centre Institute of Genetic Medicine Newcastle University Newcastle Hospitals NHS Foundation Trust Central Parkway Newcastle Upon Tyne United Kingdom NE1 4EP; ^2^ Department of Molecular Neurosciences MRC Centre for Neuromuscular Diseases UCL Institute of Neurology London United Kingdom; ^3^ Department of Neurology Copenhagen Neuromuscular Center Rigshospitalet University of Copenhagen Blegdamsvej 9 2100 Copenhagen Denmark; ^4^ Institute of Myology AP6HP, G‐H Pitié‐Salpêtrière 47‐83 boulevard de l'hôpital 75651 Paris Cedex 13 France; ^5^ The Robert Jones and Agnes Hunt Orthopaedic Hospital Oswestry Shropshire United Kingdom; ^6^ Institute of Myology Neuromuscular Investigation Center Pitié‐Salpêtrière Hospital Paris France; ^7^ Newcastle Magnetic Resonance Centre Institute of Cellular Medicine Newcastle University Newcastle upon Tyne United Kingdom

## Abstract

**Objective:**

Limb girdle muscular dystrophy type R9 (LGMD R9) is an autosomal recessive muscle disease for which there is currently no causative treatment. The development of putative therapies requires sensitive outcome measures for clinical trials in this slowly progressing condition. This study extends functional assessments and MRI muscle fat fraction measurements in an LGMD R9 cohort across 6 years.

**Methods:**

Twenty‐three participants with LGMD R9, previously assessed over a 1‐year period, were re‐enrolled at 6 years. Standardized functional assessments were performed including: myometry, timed tests, and spirometry testing. Quantitative MRI was used to measure fat fraction in lower limb skeletal muscle groups.

**Results:**

At 6 years, all 14 muscle groups assessed demonstrated significant increases in fat fraction, compared to eight groups in the 1‐year follow‐up study. In direct contrast to the 1‐year follow‐up, the 6‐min walk test, 10‐m walk or run, timed up and go, stair ascend, stair descend and chair rise demonstrated significant decline. Among the functional tests, only FVC significantly declined over both the 1‐ and 6‐year studies.

**Interpretation:**

These results further support fat fraction measurements as a primary outcome measure alongside functional assessments. The most appropriate individual muscles are the vastus lateralis, gracilis, sartorius, and gastrocnemii. Using composite groups of lower leg muscles, thigh muscles, or triceps surae, yielded high standardized response means (SRMs). Over 6 years, quantitative fat fraction assessment demonstrated higher SRM values than seen in functional tests suggesting greater responsiveness to disease progression.

## Introduction

Limb girdle muscular dystrophy type R9 (LGMD R9) is an autosomal recessive disease caused by mutations in the *Fukutin‐related protein* gene (*FKRP*).^1^ LGMD R9 is one of the most common limb girdle muscular dystrophies in Northern and Central Europe, with a prevalence of 1 in 230,000^2^ in the UK and 1 in 54,000 in Norway.^3^ Most patients with LGMD R9 share a common homozygous founder mutation (c.826C>A, p.Leu276Ile) and a relatively mild phenotype compared to the compound heterozygous form.^4^, ^5^ LGMD R9 presents with slowly progressive muscular weakness, variably affecting skeletal, respiratory and cardiac muscles.^6^, ^7^ Onset and severity of symptoms are highly heterogeneous. Putative therapeutic approaches, including gene therapy,^8^, ^9^ immunomodulation^10^, ^11^ and targeting glycosylation of *α*‐dystroglycan,^12^ are currently being evaluated. As therapeutics are developed, it is important to establish sensitive outcome measures for clinical trials. The most commonly used outcome measures in clinical trials of muscular dystrophies are functional assessments.^13^ Functional tests are clinically relevant and are considered important by regulatory agencies in therapeutic trial design for muscular dystrophies.^5^ Due to the slow progression of LGMD R9, standard strength and functional measures of skeletal muscle were unable to show a significant difference over 1 year.^14^ Willis et al. investigated the use of the 3‐point Dixon magnetic resonance imaging (MRI) technique, which allows to calculate fat fraction using acquisitions with separated water and fat images.^15^ The percentage of fat replacement can be used as a biomarker of disease progression within the LGMD R9 cohort. The study found that 9/14 muscles of the legs demonstrated a significant increase in fat replacement over 12 months; the Dixon technique was more sensitive to disease progression than functional assessments.^14^, ^16^

The purpose of this study was to investigate various outcome measures in the previously recruited LGMD R9 cohort over 6 years. Fat fraction in skeletal muscle and functional assessments were evaluated to learn more about the natural history of the disease, to assess the progression of selective muscle pathology, to identify useful functional and imaging outcome measures and to inform future trial design.

## Methods

All available participants from the original study, running from 2009 to 2011,^14^ were approached to undergo further functional assessments and quantitative MRI measurements as close to 6 years after the original measurements as possible. In the original study, the initial recruitment was from four sites (Newcastle upon Tyne, UK; London, UK; Copenhagen, Denmark; and Paris, France). The inclusion and exclusion criteria of the original study included: homozygous for the c.286C>A, p. L276I mutation, ambulant without support for greater than 50 m, no ventilator requirement, ability to lie supine, and no contraindications to MRI.

As the study by Willis et al. was limited to a 1‐year follow up, new ethical approvals were obtained at the four centers. The study complied with the Declaration of Helsinki and received ethics and R&D approval at all sites involved.

Participants underwent a series of functional assessments, including: 6‐min walk test (6MWT),^13^, ^17^ 10‐m walk or run,^18^ timed up and go,^19^ stair ascend, stair descend and chair rise. Myometry was performed on the dominant side using a hand‐held myometer (Citec or Microfet) assessing: knee flexion, knee extension, hip abduction, hip adduction, and ankle dorsiflexion. Forced vital capacity (FVC) was measured and expressed as a percentage of predicted value for height. Interobserver consistency was ensured using standardized manuals and equipment, and training via teleconference. To incorporate participants unable to perform assessments, results were expressed as a velocity (msec^−1^ or stairs/sec). For the 10‐m walk or run, the number of meters was divided by the time taken, the number of stairs (four) was divided by stair ascend and descend times, and the 6 m were divided by timed up and go test time. Cardiac function was monitored as part of clinical care, but this was not systematically assessed across all centers for the purpose of this study.

### Recruitment

At baseline assessment, the median age of the participants (*n* = 23) was 39.1 years (interquartile range (IQR) 27.4–50.6). Median length of follow‐up was 6.1 years (IQR 5.8–6.1). In the original study by Willis et al., participants were required to be ambulant with an ability to walk over 50 m^7^, ^14^: by 6 years, six participants were nonambulant. At baseline, no participants received noninvasive ventilation (NIV), by 6 years five participants required NIV overnight. Seven participants received cardioactive medication at baseline increasing to thirteen at 6 years.

### MRI acquisition

All scans were performed on 3T scanners (Philips Achieva, Siemens TIM Trio, and Skyra) using surface coil arrays. Three‐point Dixon images were acquired using a spoiled gradient echo sequence, Newcastle and London used 2D TR/TE = 100/3.45,4.6,5.75 msec, flip angle of 10 degrees, 10 slices of 10 mm slice thickness with a 5 mm gap. Paris used a 3D sequence with TR/TE = 10/2.75,3.95,5.15 msec, flip angle of 3 degrees, 64 slices of 5 mm thickness; Copenhagen as per Paris, but with 36 slices per 3D acquisition, and 2‐point Dixon with correction for homogeneity of B_0._ 15 The data were processed off‐line to produce separate fat and water images.^15^, ^20^ Quantitative fat fraction maps were produced by expressing the fat signal as a percentage of the total signal per voxel. Phantom measurements and healthy volunteer images were acquired at each site prior to the studies. To ensure consistency of positioning among sites, acquisitions in the legs were positioned with the patella anterior and the lower leg images centered by locating the broadest region of the lower leg and recording the distance from the lower border of the patella. Positioning of the thigh images was ensured by locating the superior border of the patella and acquiring one‐third between this and the anterior superior iliac spine. A matrix of 160 × 160 interpolated to 256 × 256, field of view (FOV) 200 × 200 mm was used with each leg imaged separately. The Paris site was able to scan both left and right legs at the same resolution using FOV 448 × 244 mm.

### Data analysis

Both functional assessments and MRI data were anonymized and transferred to Newcastle. Regions of interest (ROIs) were defined using the imaging software ‘Image J'.^21^ Two observers drew ROIs independently around the selected muscle groups at a single level. Data from the left and right legs were combined by averaging, as were the results from the two analysts. Interobserver comparison was performed using a Bland Altman analysis.

The ROIs were used to calculate mean fat fraction and muscle cross sectional area (CSA). Contractile cross‐sectional area (c‐CSA) was calculated by multiplying the CSA by one minus the fat fraction, representing the remaining muscle content of the ROI.

To reflect the fact that muscles function as groups, composite group fat fractions were calculated as area‐weighted averages. Composite muscle groups included: all muscles of the thighs, all muscles of the lower legs, quadriceps (rectus femoris (RF), vastus lateralis, vastus medialis), hamstrings (semimembranosus, semitendinosus, biceps femoris short head (BFSH), biceps femoris long head), and the triceps surae (lateral gastrocnemius, medial gastrocnemius, soleus).

Following analysis of the fat fraction results, a post hoc group of potential ‘target muscles' for use in future clinical trials was identified. The five muscle groups were picked to demonstrate progression over 6 years with the highest standardized response means (SRM ≥0.91) that had also demonstrated significant change at 1 year in the Willis study^14^ (soleus was excluded as it had not shown significant change at 1 year), allowing interim analysis. These muscles are reported as an additional composite group using area‐weighted fat fraction averaging in Tables 2 and 3 (“averaged target muscles”). The averaged target muscle group consisted of the vastus lateralis, gracilis, sartorius, medial gastrocnemius, and lateral gastrocnemius.

In order to identify whether there were particular individuals who had very low progression of fat replacement of most muscle groups over the 6 years, we identified for each participant, how many muscle groups they had that had a fat fraction of less than 20% fat at baseline and progressed by less than 20% fat over 6 years.

### Statistical analysis

Statistical analysis was performed using SPSS v24, with data presented as median and range unless otherwise indicated. Statistical significance was calculated using the Wilcoxon test for nonparametric data. For nonparametric testing of the chair rise time, where a participant was unable to perform a timed functional assessment, a value greater than the longest possible result (10,000s) was used to provide correct ranking. Statistical significance was taken to be *P* < 0.05.

Standardized response means (SRM) were calculated for all outcome measures by taking the average of the paired difference over the 6 years divided by the standard deviation of these differences. A high SRM (>0.8) implied that a test had a high level of responsiveness to changes in value.^22^

### Data availability statement

The anonymized MRI measurements, physical function tests and clinical characteristics will be made available via the Dryad data repository (Data available from the Dryad Digital Repository: https://doi.org/10.5061/dryad.f3r6799).

## Results

### Functional assessments

All of the timed assessments of skeletal muscle demonstrated a significant change over 6 years (Table 1). The 6MWT and 10‐m walk or run tests had significant change with *P* ≤ 0.001 and high SRMs over 6 years (−0.85 and −1.02 respectively). Of the myometry assessments, only measurement of hip adduction decreased significantly over the period of follow‐up (median baseline 6.1 kg, median 6 years 4.2 kg, *P* = 0.02). The annual median FVC decline in a sitting position was −2.6% (−5 to 1.8%), with −1.9% (−7.3 to 1.5%) median annual decline when in the supine position. Both measures of FVC also had high SRM (−1.29 and −1.06 respectively).

**Table 1:** The median change in functional assessments over the follow‐up of 6 years

Functional assessment	Median baseline (range)	Median 6 years (range)	*P* value	SRM
Forced vital capacity sitting (%)^c^	77 (55–94)	64 (31–86)	0.001^a^	−1.29
Forced vital capacity lying (%)^d^	70 (36–90)	54 (21–84)	0.002^a^	−1.06
Hip flexion (kg)	7.7 (0.0–36.8)	6.4 (0.0–24.3)	0.13	−0.35
Hip adduction (kg)	6.1 (0.7–26.7)	4.2 (0–23.9)	0.02^a^	−0.59
Hip abduction (kg)	8.3 (0.6–25.0)	7.2 (2.2–25.3)	0.76	−0.01
Knee extension (kg)	11.1 (2.0–40.3)	12.0 (1.5–39.1)	0.15	−0.26
Knee flexion (kg)	8.4 (0.9–30.0)	4.8 (0–37.9)	0.21	−0.12
Ankle dorsiflexion (kg)	14.7 (2.5–38.6)	13.9 (2.4–26.3)	0.07	−0.42
Six‐minute walk (meters)	391 (67–625)	286 (0–750)	0.001^a^	−0.85
Timed up and go velocity (msec^−1^)^e^	0.5 (0.0–1.7)	0.3 (0.0–1.6)	0.007^a^	−0.48
Ten‐meter walk or run velocity (msec^−1^)^e^	1.2 (0.5–4.4)	0.8 (0.0–3.9)	<0.001^a^	−1.02
Stair ascent velocity (steps/sec)^e^	0.7 (0.0–4.4)	0.4 (0.0–3.0)	0.001^a^	−0.46
Stair descent velocity (steps/sec)^e^	1.2 (0.0–4.4)	0.4 (0.0–3.3)	0.008^a^	−0.47
Chair rise (sec)^b^, ^c^	2.6 (0.3 to ∞)	9.8 (0.5 to ∞)	0.001^a^	N/A

*P* values calculated using Wilcoxon nonparametric rank signed test. SRM – standardized response mean.

a *P* value <0.05.

b Participants unable to perform the chair rise were given a value of 10,000 (effectively infinity) for the purposes of the Wilcoxon nonparametric rank signed test.

c Paired values available in 17 participants.

d Paired values available in 16 participants.

e Paired values available in 22 participants.

Two participants improved their speed for the 10‐m walk or run assessment over 6 years, however, both reduced in their 6MWT distance. Over 6 years, the 6MWT distance significantly declined, with six participants becoming nonambulant. Four participants improved their distance (mean increase 68.8 m ± 50.3). These four subjects showed a fat fraction increase of at least 1% in the majority of the twenty muscle groups and composite groups studied (15/20 groups for one patient, 19/20 groups for two patients and all groups for one patient). Looking at the increase in fat fraction of the two participants that improved in 10‐m walk or run test velocity, one had >1% increase in all muscles, the other had only one muscle with >1% increase.

### Quantitative fat fraction

**Figure 1:**
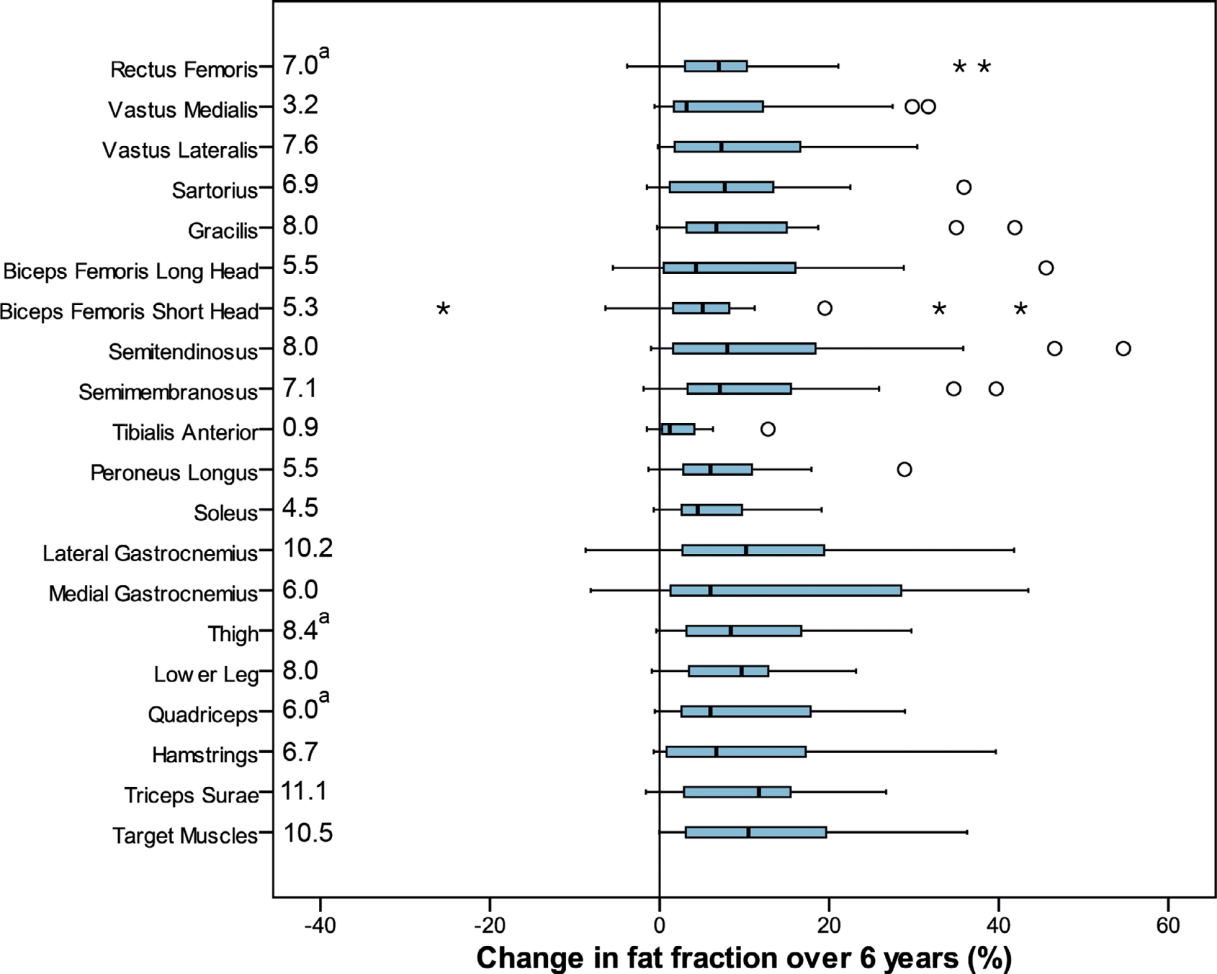
Box plot showing the change in fat percentage from baseline to 6 years. The blue bars show the interquartile range and the median. The lines show the range of the data. Outliers one to three times the interquartile range are marked as circles. Outliers greater than three times the interquartile range from the median are shown as individual asterisks. The median change is shown for each muscle group on the left. Note that the median change will not be equal to the difference in the median baseline and 6 year medians given in Table 2. a Due to difficulties in ROI placement in the rectus femoris muscle, the fat fractions for two participants were excluded for this muscle group, and no composite results calculated where appropriate (n=21).

Interobserver consistency of ROI analysis was assessed using the Bland‐Altman analysis. The observers had a mean difference in fat fraction of 0.05%. The 95% limits of interobserver agreement ranged from the BFSH at 10.46% down to the soleus at 1.12% (Table S1). The wide limits of agreement for BFSH are likely due to the small size of the muscle in the transverse plane. One of the next widest limits of agreement was found in the RF muscle, where difficulties defining the borders were complicated by the high level of fat replacement (Fig. 1). The values of the RF muscle from two participants were excluded from analysis due to the high levels of discrepancy between observers in ROI placement. The interobserver variability in fat fraction demonstrated in the BFLH, semitendinosus and semimembranosus muscles may be caused by high levels of fat replacement at follow‐up, making recognition of ROI borders difficult.

**Figure 2:**
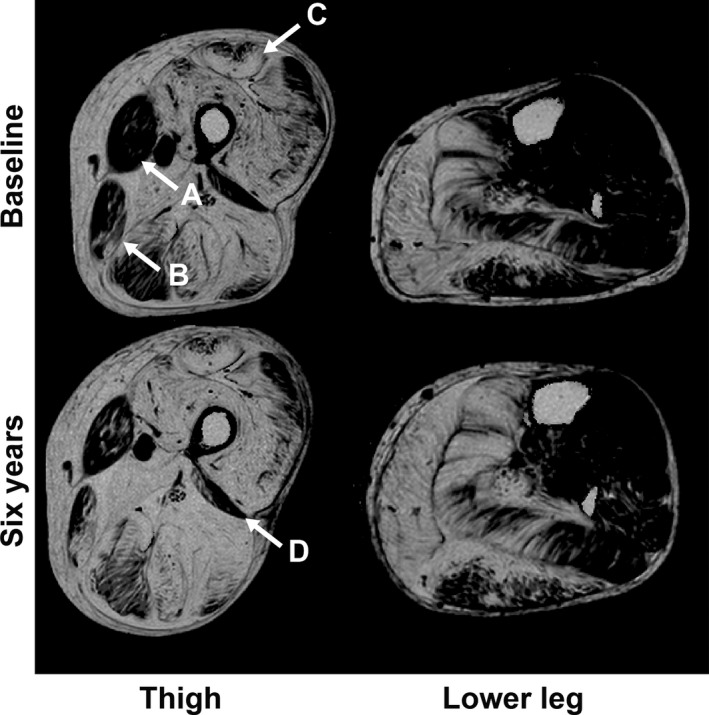
Images showing the change in fat replacement over 6 years. Fat fraction maps acquired from the left thigh and lower leg at baseline and 6‐year follow‐up (0–100% scale). Progression of fat replacement was visible in almost all muscles, with changes most noticeable in the muscles relatively spared at baseline, such as the Sartorius (white arrow A ‐ in this participant fat fraction increased from 21.3% to 34.2%) and the gracilis (white arrow B ‐ increased from 40.3% to 58.7%) muscles. As indicated by the white arrow (C), fat replacement began at the borders of the rectus femoris muscle at both baseline and 6 years, which caused difficulties in ROI placement. In this participant, the fat fraction was 78.9% at baseline increasing to 81.9% at 6 years. The shape and size of the biceps femoris short head muscle also caused difficulties in ROI placement as demonstrated by the white arrow (D).

**Figure 3:**
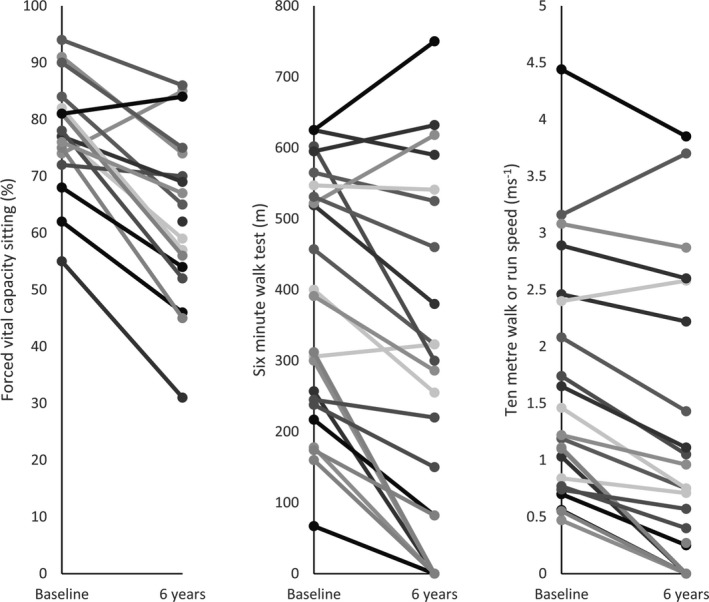
Line graphs demonstrating individual participant progression in functional tests from baseline to 6 years. The functional assessments with the highest SRM values were selected to compare individual progression over time. On the 6‐min walk test, there was no clear cut‐off for predicting loss of ambulation, 6/9 participants who walked ≤300 m became nonambulant by 6 years. Two participants improved on both the 10‐m walk or run test and sitting forced vital capacity.

**Figure 4:**
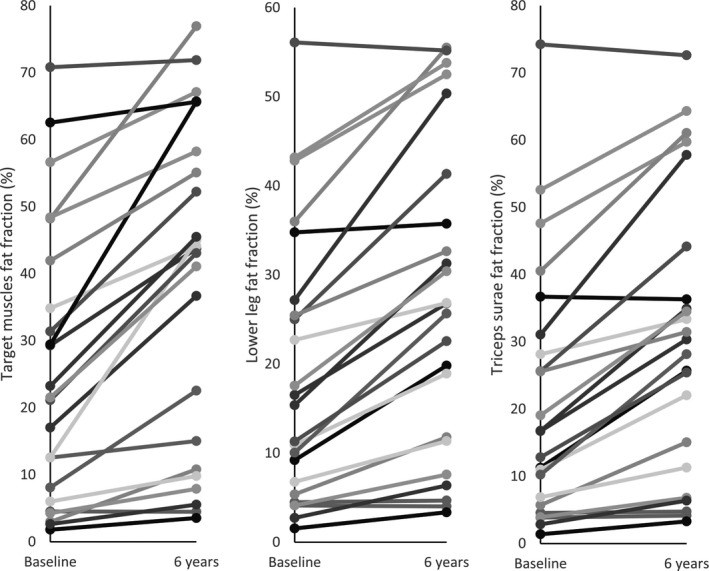
Line graphs demonstrating individual participant progression in fat fraction from baseline to 6 years. The composite muscle measures with the highest SRMs were selected to compare individual progression over time. Quadriceps and thigh measures were excluded as these were not available in all participants due to difficulties in ROI placement in the rectus femoris. The individual lines suggest there is only one participant who does not progress over the 6 years and six participants who have little infiltration at baseline and increase relatively slowly over the 6 years.

Over the 6 years, all 14 muscle groups demonstrated a significant increase in percentage of fat replacement (Table 2 and Fig. 2). The highest median percentage of fat replacement at baseline was in the biceps femoris long head muscle (BFLH) at 69.4%, increasing to 78.6% at 6 years. The tibialis anterior muscle (TA) was least affected at baseline, median 5.2%, and at follow‐up showed a median of 7.1%. The TA also had the smallest median change over the 6‐year period (Fig. 2). LGMD R9 demonstrated phenotypic variability between individuals: changes in selected functional assessments over time are shown for individuals in Figure 3. The source data for these and all other measures are available in the Dryad data repository. When considering the most complete composite muscle groups with the highest SRM, there were small numbers of participants whose fat fraction did not increase over the 6 years (Fig. 4).

**Table 2:** Median muscle fat fractions at baseline and 6‐year follow up

Muscle group	Median baseline (range), %	Median 6 years (range), %	*P* value	SRM
Rectus femoris^b^, ^c^	10.8 (0.9–81.9)	19.9 (0.4–92.2)	<0.001^a^	0.90
Vastus medialis	21.0 (1.1–86.6)	44.4 (2.5–87.9)	<0.001^a^	0.83
Vastus lateralis^c^	13.7 (1.5–65.2)	36.1 (2.8–81.7)	<0.001^a^	0.92
Sartorius^c^	20.9 (1.7–89.8)	34.2 (5.4–88.3)	<0.001^a^	0.98
Gracilis^b^	18.4 (3.7–81.0)	32.4 (4.5–90.2)	<0.001^a^	1.04
Biceps femoris long head^c^	69.4 (2.2–97.7)	78.6 (4.8–100)	0.001^a^	0.81
Biceps femoris short head	21.3 (3.2–94.5)	32.5 (4.6–84.3)	0.002^a^	0.53
Semitendinosus^c^	35.6 (2.1–100)	69.6 (4.1–100)	<0.001^a^	0.83
Semimembranosus^c^	25.7 (0.5–95.1)	53.4 (4.6–99.2)	<0.001^a^	0.90
Tibialis anterior	5.2 (0.9–25.3)	7.1 (1.1–27.1)	0.002^a^	0.67
Peroneus longus	15.8 (3.0–46.2)	18.0 (4.6–65.2)	0.001^a^	0.88
Soleus	7.5 (1.8–67.4)	16.7 (3.1–70.1)	<0.001^a^	1.17
Lateral gastrocnemius^c^	19.4 (0.9–76.1)	35.8 (3.1–75.2)	<0.001^a^	0.91
Medial gastrocnemius^c^	19.9 (1.1–91.8)	48.0 (2.8–84.2)	<0.001^a^	0.95
Averaged thigh^b^	30.8 (2.1–76.2)	47.3 (4.2–82.1)	<0.001^a^	1.20
Averaged lower leg	15.4 (1.6–56.1)	26.8 (3.4–55.5)	<0.001^a^	1.28
Averaged quadriceps^b^	23.0 (1.3–67.6)	40.4 (2.6–79.8)	<0.001^a^	1.05
Averaged hamstrings	45.0 (2.8–97.8)	60.4 (4.7–98.7)	<0.001^a^	0.93
Averaged triceps surae	16.7 (1.4–74.2)	30.3 (3.3–72.6)	<0.001^a^	1.25
Averaged target muscles	21.5 (1.8–70.8)	43.8 (3.6–76.9)	<0.001^a^	1.23

*P* values calculated using Wilcoxon nonparametric rank signed test. SRM – standardized response mean.

a *P* value<0.05.

b Due to difficulties in ROI placement in the rectus femoris muscle the fat fractions for two of the participants were excluded for this muscle group (*n* = 21). As the rectus femoris measures are excluded from two participants, it is not possible to create composite measures (thigh and quadriceps) involving this muscle for these participants. The thigh and quadriceps measures are based on 21 participants.

c Exhibited a significant increase in fat fraction over a 1‐year period taken from Willis et al.^7^

The CSA and c‐CSA results are presented in Table 3. The CSA did not significantly decline in any of the muscles over the period of the study. The c‐CSA was significantly decreased in 8 of the 14 individual muscles and in all of the averaged muscle groupings (Table 3). The median increase in fat fraction per year from this study and the 12‐month Willis study^13^ is given in Table S2, together with a correlation between the fat fraction change over 12 months and 6 years: correlation indicates where the short‐term measurement may be predictive of long‐term change. Significant correlation was found for the semitendinosus, medial gastrocnemius, peroneus longus, soleus, vastus medialis, and tibialis anterior.

**Table 3:** Median cross‐sectional area (CSA) and contractile cross‐sectional area (c‐CSA) at baseline and 6 years

Muscle group	CSA baseline (mm^2^)	CSA 6 years (mm^2^)	c‐CSA baseline (mm^2^)	c‐CSA 6 years (mm^2^)	*P* value	c‐CSA SRM
Rectus femoris^b^	343	344	260	232	0.009^a^	−0.51
Vastus medialis	720	932	428	389	0.11	−0.15
Vastus lateralis	1639	1517	1195	1025	0.003^a^	−0.62
Sartorius	402	425	346	231	0.06	−0.41
Gracilis	344	309	168	154	0.003^a^	−0.64
Biceps femoris long head	1012	911	316	145	0.02^a^	−0.49
Biceps femoris short head	153	157	110	81	0.63	−0.16
Semitendinosus	687	679	316	206	0.002^a^	−0.71
Semimembranosus	685	671	410	301	0.04^a^	−0.37
Tibialis anterior	568	569	536	537	0.67	−0.20
Peroneus longus	578	546	464	436	0.09	−0.29
Soleus	1803	1635	1512	1430	0.09	−0.35
Lateral gastrocnemius	553	523	435	317	0.04^a^	−0.39
Medial gastrocnemius	1243	993	879	516	0.02^a^	−0.53
Averaged thigh^b^	6024	6071	3288	3087	0.006^a^	−0.55
Averaged lower leg	4263	4264	3637	3365	0.01^a^	−0.52
Averaged quadriceps^b^	3078	3103	2017	1577	0.01^a^	−0.44
Averaged hamstrings	2322	2347	1101	842	0.007^a^	−0.63
Averaged triceps surae	3272	3113	2834	2469	0.009^a^	−0.53
Averaged targeted muscles	4211	3803	2745	2248	0.005^a^	−0.67

All *P* values quoted refer to c‐CSA. There are no significant changes in CSA between baseline and 6 years.

SRM, standardized response mean.

a Denotes a *P* value <0.05.

b Due to difficulties in ROI placement in the rectus femoris muscle, the fat fractions for two of the participants were excluded for this muscle group (*n* = 21).

The 10‐m walk or run test velocity and the 6MWT had several significant negative correlations with the fat fraction of most muscle groups at baseline and 6 years (Tables S3 and S4. These were strongest in the composite muscle groups (such as the thigh (*r* from −0.83 to −0.91, *P* < 0.001), hamstrings (*r* from −0.71 to −0.81, *P* < 0.001), and quadriceps (*r* from −0.76 to −0.86, *P* < 0.001)) (Tables S3 and S4). Changes in fat fraction and the 6MWT across 6 years correlated moderately in the soleus (*r* = −0.6, *P* < 0.05), RF (*r* = −0.53, *P* < 0.05) and weakly in some of the composite muscle groups: the thigh (*r* = −0.47, *P* < 0.05), quadriceps (*r* = −0.46, *P* < 0.05), and triceps surae (*r* = −0.45, *P* < 0.05). Changes in fat fraction and the 10‐m walk or run test velocity across 6 years correlated weakly only in the soleus muscle (*r* = −0.52, *P* < 0.05).

Five individuals had less than 20% fat replacement at baseline, and then progressed by less than 20% over 6 years in all muscle groups. For one further individual, this was true for eleven muscle groups (Table 4). This group of six subjects whom we termed “slow progressors”, were significantly younger than the other 17 participants (median age at baseline 23.5 vs. 43.0 years, *P* < 0.001).

**Table 4:** Comparison of clinical characteristics of the LGMD2I cohort with individual fat fraction changes over 6 years from baseline

Part. No.^a^	BL age and gender	CM/NIV	BL 6MWT (m)	BL 10 m walk or run	BL target Muscle FF (%)^b^	∆Target muscle FF(%)^b^	BL Triceps Surae FF(%)	∆TricepSurae FF*(%)*
11	58F	Y/Y	217^d^	0.7	29.4^e^	36.2	11.3	14.4
12	41F	Y/N	400^d^	1.5	12.6	32.4	11.0	11.1
6	22M	Y/N	160^c^, ^d^	0.6	48.2	28.7	40.5	20.5
15	31F	N/N	519	1.7	23.3	22.3	31.1	26.7
21	44F	Y/N	245	0.7	21.1	22.0	12.8	12.6
7	27F	N/N	602	1.7	31.4	20.8	25.6	18.5
8	33M	N/N	625	2.5	17.1	19.6	16.8	18.1
23	43F	N/N	391	1.2	21.5	19.5	19.1	15.5
1	62M	Y/Y	257^c^, ^d^	1.0	29.3	14.5	16.7	13.6
17	27F	Y/N	531	2.1	8.1	14.4	10.2	17.9
20	39F	N/N	174	0.0	42.0	13.1	25.6	5.9
2	64M	Y/N	178^c^, ^d^	0.5	56.6	10.5	47.6	12.1
9	38M	Y/N	300^c^, ^d^	1.1	48.4	9.8	52.6	11.7
5	56F	Y/N	306	0.8	34.9	9.3	28.2	5.2
13	22M	N/Y	312^c^, ^d^	1.1	3.0	7.8^e^	5.7	9.3
19	42F	N/N	547	2.4	6.0	3.8^e^	6.9	4.4
16	29F	N/N	522	3.1	4.3	3.6^e^	3.9	2.8
4	47M	Y/Y	67^c^, ^d^	0.6	62.6	3.1	36.7	‐0.4
22	27F	Y/Y	595	2.9	2.7	2.9^e^	2.9	3.5
3	58F	Y/N	457	1.2	12.6	2.5	3.9	0.3
18	18M	N/N	625	4.4	1.8	1.7^e^	1.4	1.9
14	51M	Y/N	238	0.8	70.8	1.1	74.2	‐1.6
10	8M	N/N	565	3.2	4.5	0.0^e^	4.6	0.2

BL, Baseline; CM, cardiomyopathy; NIV, noninvasive ventilation; SP, slow progressor; LL, lower leg; 6MWT, 6‐min walk test; 10MWR, ten‐meter walk or run.

a Refers to participant number in the Dryad repository.

b “Target muscles” is a composite group consisting of vastus lateralis, gracilis, sartorius, medial gastrocnemius and lateral gastrocnemius with progression at 1 year and high SRM.

c Subject has lost ambulation by 6‐year follow‐up.

d Participant cannot perform chair rise at 6‐year follow‐up.

e Slow progressor, defined as: the majority of muscle groups with <20% FF baseline and <20% increase over 6 years.

To provide a sense of the heterogeneity in fat fraction changes between individual subjects, we provide Table 4 which includes individual details of clinical characteristics, baseline values for the 6MWT and 10‐min walk or run, and baseline and 6‐year changes in fat fraction for the target and triceps surae muscle groups. These muscle groups were chosen since they are available for all subjects and had high SRMs at 6 years. There is also information on whether these subjects lost ambulation at 6 years and/or were unable to perform the chair rise. The participants are ordered in decreasing order of fat fraction increase in the target muscles across 6 years (changes ranged from 36.2% to 0.0%). Similar individual results for other muscle groups can be obtained within the Dryad data repository.

## Discussion

As putative therapies for LGMD R9 are evaluated,^8^, ^10^, ^12^ sensitive longitudinal outcome measures are required.^5^ This study built upon our previous 1‐year study,^14^ representing the longest and largest multicenter study of LGMD R9 to date. These results provide support for 3‐point Dixon technique as an outcome measure over both 1 and 6 years and identified functional assessments significantly declining over a 6‐year period, whereas significant decline could not be detected in functional assessments over 1 year.^14^

### Functional assessments

A recent workshop assessing trial readiness in LGMD R9 highlighted the need for MRI alongside other functional assessments, with emphasis on measuring movements that affected quality of life.^5^ The results of this study and Willis et al. suggested that the Dixon technique can reliably detect changes in leg muscles over 1 and 6 years in LGMD R9. None of the ambulatory skeletal muscle functional assessments demonstrated a significant difference over 1 year.^14^ Our study suggests that, over 6 years, all timed muscle function tests demonstrated disease progression, though of the hand‐held myometry tests, only hip adduction strength was significantly decreased. The 6MWT and 10‐m walk or run tests had high SRMs over 6 years; these assessments are therefore worth measuring over the longer time period, even though insensitive over 12 months. There was a degree of individual variability in functional assessments at 6 years with two participants increasing their velocity on the 10‐m walk or run but reducing their 6MWT distance. The differences between the fat fraction increase and the results of the 6MWT and 10‐m walk or run test support MRI as a more meaningful outcome measure and highlight the participant‐dependent factors of functional testing (such as effort or fatigue). In contrast to Duchenne muscular dystrophy (DMD) studies,^23^ there was no clear cut‐off in distance walked at baseline which could predict nonambulation in the LGMD R9 cohort at 6 years.

The only myometry measurement which demonstrated a significant decline over 6 years was hip adduction. This suggests limited sensitivity to change in a slowly progressing disease. All timed functional assessments demonstrated a significant decline, with only the 6MWT and the 10‐m walk or run velocity having SRM values >0.5 (Table 1). In DMD, myometry measurements may be predictive of decline, though they are not always significant even over 2 years.^23^ The timed tests lack the sensitivity to detect the slowly progressive weakness of LGMD R9 over 1 year.^14^

FVC was the only functional assessment which changed significantly in 1 year and over a 6‐year period (Table 1).^14^ The median annual decline is less than reported elsewhere, perhaps explained by the wide age‐range of our cohort.^14^, ^24^ Respiratory muscle involvement is only indirectly linked to ambulatory skeletal muscle involvement. The rate of annual median FVC decline was not clinically significant but likely to have a cumulative effect.

As a potential clinical trial outcome measure, the 6MWT and 10‐m walk or run were the only functional timed assessments relating to skeletal muscle demonstrating a significant difference over 6 years with all participants included at baseline. The 6MWT, 10‐m walk or run, timed up and go, chair rise, stair ascend, and stair descend tests are important to include as outcome measures in future trials investigating LGMD R9 cohorts. Our study demonstrated that quantitative MRI was more sensitive within this cohort, most likely due to the slow progression of the LGMD R9 phenotype.

### Fat replacement as an outcome measure

The fat fraction has been established as a sensitive measure of disease progression in muscular dystrophies.^14^, ^25^, ^26^, ^27^, ^28^, ^29^, ^30^, ^31^, ^32^, ^33^ The fat fraction of all 14 muscles assessed significantly increased over 6 years.

For every muscle group, the SRM for the c‐CSA is of smaller magnitude (and the *P* value less significant) than for the respective fat fraction alone, indicating that the variability in the cross‐sectional areas within the group outweighs the small yet progressive changes in fat fraction. LGMD R9 has been associated with a lower c‐CSA compared to controls, but preserving the ratio between the c‐CSA and torque^34^: it is not possible to confirm this relationship in the present data due to different physical function tests used. c‐CSA has been shown to correlate to functional measures of strength in other muscular dystrophies.^34^, ^35^ In this case, c‐CSA is a less sensitive endpoint than fat fraction alone.

To maximize the discriminant power of quantitative MRI as a biomarker for therapeutic studies, it was useful to select muscle groups for analysis. Variability of muscle involvement and ability to demonstrate a significant change over short intervention periods are important. Willis et al. identified nine muscle groups whose fat fractions increased significantly over a 12‐month period.^14^ In the upper leg, the vastus lateralis, sartorius and gracilis muscles were easily identifiable at baseline and follow‐up. In the lower leg, Willis et al. suggested that the medial gastrocnemius muscle should be used for analysis.^14^ Both at baseline and 6 years, the medial and lateral gastrocnemii muscles had similar levels of fat fraction with little difference in variability (Table 2). The results showed several highly significant p values coupled with high SRM values, suggesting that to maximize power in a trial the following muscles should be targeted in the thigh: vastus lateralis, gracilis, and sartorius. In the calves, both of the gastrocnemii muscles should be included for analysis. Other thigh muscles, including the BFLH, semitendinosus and semimembranosus, would not be suitable in spite of high SRM values. These muscles showed high fat fractions at baseline, making it likely that therapeutic response would be small. High levels of fat at baseline also increased the difficulty and reliability of ROI placement. Fat fractions of the vastus medialis, BFSH and remaining calf muscles (peroneus longus, soleus, and TA) were less suitable endpoints due to lack of significant differences at 1 year in the Willis study,^14^ reducing the usefulness of any interim analysis. Other composite muscle groups such as the averaged thigh, averaged lower leg and averaged triceps surae muscles had high SRM values, similar to the value of the target muscle group. Therefore, these composite measures may have utility as a powerful outcome measure.

Looking at the characteristics of the individual subjects using the target muscle and triceps surae groups as exemplars (Table 4), those at the bottom of the table with least change in fat fraction tended to have the lowest baseline fat fractions, though there were exceptions. Likewise, there was no particular value of baseline 6MWT or 10‐m walk or run that predicted greater change in this group.

It is important to acknowledge that these results may underestimate progression of the disease process with most severely affected individuals less likely to return at 6 years due to difficulties in travelling. Other limitations included that only one slice was analyzed in each subject at a predefined level whereas multislice analysis could take account of heterogeneity in disease progression. While there were many possible correlations between fat fraction calculations and functional measures, this work concentrated principally on the sensitivity of outcome measures to detect change over time. For future work, it would be appropriate to accompany MRI and functional testing with self‐reported Quality of Life assessment tools as per recent recommendations^5^: several suitable options exist including the Quality of Life in genetic Neuromuscular Disease questionnaire (QoL‐gNMD),^36^ the Activity Limitation (ACTIVLIM) questionnaire for neuromuscular disorders^37^ and general tools such as the Fatigue Severity Scale.^38^

This study is the longest follow‐up of a LGMD R9 cohort and demonstrated that fat fraction measurement was the most sensitive marker of disease progression over a 6‐year period. Long‐term natural history data are of value in postmarketing and long‐term surveillance in these rare diseases. Use of the Dixon technique can provide useful interim measures of disease progression not currently possible with functional testing, and can be deployed in patients unable to complete all functional tests. Our results support fat fraction measurement in LGMD R9 clinical trials as a primary outcome measure alongside functional assessments: The 6MWT and the 10‐m walk or run were the most appropriate, relevant functional measures for a longer therapeutic trial. This study provides direction for clinical trial development and outcome measures for powering future randomized controlled trials into LGMD R9.

## Author Contributions

APM and KGH were involved in the design and conceptualization of the study; analyzed the data and drafted the manuscript for intellectual content. JM, JD, TS, TW, MH, MJ, AM, ME, LL, and JYH were involved in data collection, drafting and revision of manuscript. JT, JV, CS, SW, TY PC, and VS conceptualized the study and were involved in the drafting and revision of manuscript.

## Conflict of Interest

APM, JM, JD, TS, CS, SW, MH, LL, JVH, and PC report no conflict of interest.

Professor Willis has served on advisory boards for PTC pharmaceuticals, Genzyme Sanofi, Sarepta and Biogen and has received honorariums for lectures and symposium from PTC pharmaceuticals, Genzyme Sanofi and Biogen.

Yousry has received honoraria and travel expenses for advisory committee work from Bayer Schering, Biogen Idec, and Novartis; and research grants (held by University College London) from Biogen Idec, GlaxoSmithKline, Novartis, and Schering AG for analysis of data from MS trials.

James performs consultancy work (training physiotherapists) for: Roche, Pfizer, PTC, Summit, Sarepta, Santhera, Italfarmaco, Amicus and has participated in advisory boards for PTC Therapeutics.

Mayhew performs consultancy work (training physiotherapists) for: Roche, Avexis, Biogen, Pfizer, PTC, Summit, Sarepta, Santhera, Italfarmaco, Amicus, Tamoxifen noncommercial study for DMD and has participated in advisory boards for: PTC, AMO Pharma, Roche, Summit, Biogen, Modus, Novartis.

Eagle performs consultancy work for: Biomarin, GSK, Prosensa, Italfarmaco, Amicus, Capricor, Catabasis, Eli Lilly, Fibrogen, MNK, PTC, Santhera, Solid, Summit, Sarepta, Theracon, Reveragen and Wave.

Thornton has received research support from GlaxoSmithKline, Medtronic, and Siemens.

Vissing has received research and travel support, and speaker honoraria from Sanofi Genzyme, Ultragenyx Pharmaceuticals, Santhera Pharmaceuticals and aTyr Pharma, and served as consultant on advisory boards for Sanofi/Genzyme, aTyr pharma, Ultragenyx Pharmaceuticals, Santhera Pharmaceuticals, Sarepta Therapeutics, Audentes Therapeutics and Stealth Biotherapeutics within the last 3 years.

Hollingsworth reports grants from the United Kingdom Medical Research Council, Diabetes UK, the European Union (H2020, 667078) and the Newcastle Healthcare Charity, consultancy for Summit pharmaceuticals and trial support from ImagingDMD outside the submitted work. All reimbursements were received by Newcastle University, no personal benefits were received.

Straub has or had been a chief/principal investigator for trials sponsored by Sanofi Genzyme, GSK, Prosensa/Biomarin, Ionis Pharmceuticals, and Sarepta and a subinvestigator for many other commercial studies. He received speaker honoraria from Sanofi Genzyme and is or has been on advisory boards for Audentes Therapeutics, Biogen, Biomarin, Bristol‐Myer Squibb, Exonics Therapeutics, Italfarmaco S.p.A., Nicox, Sanofi Genzyme, Santhera Pharmaceuticals, Sarepta Therapeutics, Summit Therapeutics, Tivorsan, TrophyNOD, and Wave Therapeutics. He received funding for research collaboration with Ultragenyx and Sanofi Genzyme.

## Supporting information


**Table S1.** Interobserver consistency in individual muscles.
**Table S2.** Comparison of the rate of annual median fat fraction increase.
**Table S3.** Correlation of muscle fat fractions with the 6‐min walk results.
**Table S4.** Correlation of muscle fat fractions with the 10‐m walk or run results.
