# Supplementary material for: Natural history of limb girdle muscular dystrophy R9 over 6 years: searching for trial endpoints
Source: Ann Clin Transl Neurol. 2019 May 16;6(6):1033–45. doi: 10.1002/acn3.774 (PMC6562036; doi:10.1002/acn3.774)
Supplement: Supplementary file 1 — Table S1. Interobserver consistency in individual muscles. Table S2. Comparison of the rate of annual median fat fraction increase. Table S3. Correlation of muscle fat fractions with the 6‐min walk results. Table S4. Correlation of muscle fat fractions with the 10‐m walk or run results. [file ACN3-6-1033-s001.docx]

**Supplemental Tables and captions**

Supplemental Table 1. Inter-observer consistency in individual muscles

The bias and 95% limits of agreement for fat fraction between observers in individual muscles. Data presented as bias (mean difference) and limits of agreement (1.96 times the standard deviation of the differences between observers). The widest limits of agreement were between different observers regarding the biceps femoris short head which was difficult to identify due to the shape of the muscle. The gracilis and rectus femoris had the next widest limits of agreement at 4.44 and 3.75, respectively.

| Muscle | Bias (%) | 95% Limit of Agreement (%) |
| --- | --- | --- |
| Biceps femoris short head | 0.11 | 10.46 |
| Gracilis | -0.03 | 4.44 |
| Rectus femoris | 0.28 | 3.75 |
| Vastus medialis | 0.45 | 3.37 |
| Sartorius | 0.09 | 3.23 |
| Semitendinosus | 0.34 | 3.21 |
| Lateral gastrocnemius | -0.18 | 3.11 |
| Medial gastrocnemius | -0.53 | 3.07 |
| Semimembranosus | 0.09 | 3.04 |
| Vastus lateralis | 0.14 | 2.28 |
| Biceps femoris long head | 0.05 | 2.25 |
| Peroneus longus | 0.04 | 1.83 |
| Tibialis anterior | -0.84 | 1.24 |
| Soleus | -0.03 | 1.12 |

Supplemental Table 2. Comparison of the rate of annual median fat fraction increase

Supplemental Table 2. Rate of annual median fat fraction increase derived from the six year data compared to the twelve month data presented in Willis *et al* ^13^. The change in fat fraction over one year and six years were correlated – Pearson coefficients for significant correlations are given.

| Muscle group | Annual median change in fat fraction over six years (%) (n=23) | Annual median change in fat fraction Willis *et al.* (%) (n=32) | Correlation of change in fat fraction across one year and six year follow-up (n=20) |
| --- | --- | --- | --- |
| Lateral gastrocnemius | 1.7 | 1.0 | ns |
| Semitendinosus | 1.3 | 1.7 | 0.51^a^ |
| Gracilis | 1.3 | 1.5 | ns |
| Vastus lateralis | 1.3 | 0.7 | ns |
| Sartorius | 1.2 | 1.2 | ns |
| Semimembranosus | 1.2 | 1.8 | ns |
| Rectus femoris | 1.2 ^b^ | 0.8 | ns^c^ |
| Medial gastrocnemius | 1.0 | 1.4 | 0.63^a^ |
| Biceps femoris long head | 0.9 | 1.8 | ns |
| Biceps femoris short head | 0.9 | 1.0 | ns |
| Peroneus longus | 0.9 | 0.1 | 0.45^a^ |
| Soleus | 0.8 | 0.3 | 0.50^a^ |
| Vastus medialis | 0.5 | 0.6 | 0.46^a^ |
| Tibialis anterior | 0.2 | 0.01 | 0.52^a^ |

^a^ p < 0.05.

^b^ Due to difficulties in ROI placement in the rectus femoris muscle the fat fraction for two of the participants were excluded for this muscle group (n=21).

^c^ Due to difficulties in ROI placement in the rectus femoris muscle the fat fraction for two of the participants were excluded for this muscle group (n=18)

Supplemental Table 3. Correlation of muscle fat fractions with the 6-minute walk results

Supplemental Table 3. The correlation between the fat fraction and 6MWT is shown (i) at baseline, (ii) at six years and (iii) between the change in fat fraction and change in 6MWT across the six years. Pearson coefficients for significant correlations are given. The most significant group correlations are found in the averaged thigh, hamstrings and quadriceps.

| Muscle group | Correlation between fat fraction and 6MWT at baseline | Correlation between fat fraction and 6MWT at six years | Correlation of change in fat fraction with change in 6MWT over six years |
| --- | --- | --- | --- |
| Lateral gastrocnemius | -0.60^a^ | -0.59^a^ | ns |
| Semitendinosus | -0.81^b^ | -0.67^b^ | ns |
| Gracilis | -0.68^b^ | -0.74^b^ | ns |
| Vastus lateralis | -0.79^b^ | -0.73^b^ | ns |
| Sartorius | -0.67^b^ | -0.66^b^ | ns |
| Semimembranosus | -0.86^b^ | -0.75^b^ | ns |
| Rectus femoris^c^ | -0.70^b^ | -0.80^b^ | -0.53^a^ |
| Medial gastrocnemius | -0.58^a^ | -0.57^a^ | ns |
| Biceps femoris long head | -0.69^b^ | -0.63^a^ | ns |
| Biceps femoris short head | -0.60^a^ | -0.69^b^ | ns |
| Peroneus longus | -0.45^a^ | Ns | ns |
| Soleus | -0.49^a^ | -0.56^a^ | -0.60^a^ |
| Vastus medialis | -0.76^b^ | -0.73^b^ | ns |
| Tibialis anterior | -0.47^a^ | ns | ns |
| Averaged thigh^c^ | -0.88^b^ | -0.91^b^ | -0.47^a^ |
| Averaged lower leg | -0.61^a^ | -0.59^a^ | ns |
| Averaged quadriceps ^c^ | -0.85^b^ | -0.87^b^ | -0.46^a^ |
| Averaged hamstrings | -0.81^b^ | -0.71^b^ | ns |
| Averaged triceps surae | -0.56^a^ | -0.58^a^ | -0.45^a^ |
| Averaged target muscles | -0.75^b^ | -0.75^b^ | ns |

^a^ p < 0.05

^b^ p < 0.001

^c^ Due to difficulties in ROI placement in the rectus femoris muscle the fat fraction for two of the participants were excluded for this muscle group (n=21).

Supplemental Table 4. Correlation of muscle fat fractions with the ten meter walk or run results

Supplemental Table 4. – The correlation between the fat fraction and ten-meter walk or run is shown (i) at baseline, (ii) at six years and (iii) between the change in fat fraction and change in 6MWT across the six years. Pearson coefficients for significant correlations are given. Similar to the 6MWT, the most significant group correlations are found in the averaged thigh, hamstrings and quadriceps.

| Muscle group | Correlation between fat fraction and ten meter walk or run velocity at baseline^d^ | Correlation between fat fraction and ten meter walk or run velocity at six years | Correlation of change in fat fraction with change in ten meter walk or run velocity over six years ^c^ |
| --- | --- | --- | --- |
| Lateral gastrocnemius | -0.62^a^ | -0.64^a^ | ns |
| Semitendinosus | -0.76^b^ | -0.77^b^ | ns |
| Gracilis | -0.63^a^ | -0.74^b^ | ns |
| Vastus lateralis | -0.70^b^ | -0.71^b^ | ns |
| Sartorius | -0.61^a^ | -0.67^b^ | ns |
| Semimembranosus | -0.76^b^ | -0.77^b^ | ns |
| Rectus femoris^c^ | -0.63^a^ | -0.75^b^ | ns |
| Medial gastrocnemius | -0.59^a^ | -0.65^a^ | ns |
| Biceps femoris long head | -0.78^b^ | -0.76^b^ | ns |
| Biceps femoris short head | -0.61^a^ | -0.74^b^ | ns |
| Peroneus longus | ns | ns | ns |
| Soleus | -0.49^a^ | -0.52^a^ | -0.52^a^ |
| Vastus medialis | -0.69^b^ | -0.68^b^ | ns |
| Tibialis anterior | ns | ns | ns |
| Averaged thigh ^c^ | -0.83^b^ | -0.90^b^ | ns |
| Averaged lower leg | -0.60^a^ | -0.62^a^ | ns |
| Averaged quadriceps ^c^ | -0.76^b^ | -0.80^b^ | ns |
| Averaged hamstrings | -0.80^b^ | -0.80^b^ | ns |
| Averaged triceps surae | -0.58^a^ | -0.62^a^ | ns |
| Averaged target muscles | -0.71^b^ | -0.77^b^ | ns |

*p < 0.05

^b^ p < 0.001

^c^ Due to difficulties in ROI placement in the rectus femoris muscle the fat fraction for two of the participants were excluded for this muscle group (n=21).

^d^ Results of the ten meter walk or run test not available for one participant at baseline (n=22).
